# MDM2 turnover and expression of ATRX determine the choice between quiescence and senescence in response to CDK4 inhibition

**DOI:** 10.18632/oncotarget.3364

**Published:** 2015-01-31

**Authors:** Marta Kovatcheva, David D. Liu, Mark A. Dickson, Mary E. Klein, Rachael O'Connor, Fatima O. Wilder, Nicholas D. Socci, William D. Tap, Gary K. Schwartz, Samuel Singer, Aimee M. Crago, Andrew Koff

**Affiliations:** ^1^ The Louis V. Gerstner Graduate School of Biomedical Sciences, Sloan-Kettering Institute, Memorial Sloan-Kettering Cancer Center, New York, USA; ^2^ The Graduate Program in Biochemistry, Cellular and Molecular Biology, Weill College of Medicine, Cornell University, New York, USA; ^3^ Department of Medicine, Weill College of Medicine, Cornell University, New York, USA; ^4^ Department of Surgery, Weill College of Medicine, Cornell University, New York, USA; ^5^ Program in Molecular Biology, Memorial Sloan-Kettering Cancer Center, New York, USA; ^6^ Program in Computational Biology, Memorial Sloan-Kettering Cancer Center, New York, USA; ^7^ Department of Medicine, Memorial Sloan-Kettering Cancer Center, New York, USA; ^8^ Department of Surgery, Memorial Sloan-Kettering Cancer Center, New York, USA; ^9^ Current address: Columbia University, New York, USA

**Keywords:** CDK4 inhibitors, MDM2, ATRX, senescence, geroconversion

## Abstract

CDK4 inhibitors (CDK4i) earned Breakthrough Therapy Designation from the FDA last year and are entering phase III clinical trials in several cancers. However, not all tumors respond favorably to these drugs. CDK4 activity is critical for progression through G1 phase and into the mitotic cell cycle. Inhibiting this kinase induces Rb-positive cells to exit the cell cycle into either a quiescent or senescent state. In this report, using well-differentiated and dedifferentiated liposarcoma (WD/DDLS) cell lines, we show that the proteolytic turnover of MDM2 is required for CDK4i-induced senescence. Failure to reduce MDM2 does not prevent CDK4i-induced withdrawal from the cell cycle but the cells remain in a reversible quiescent state. Reducing MDM2 in these cells drives them into the more stable senescent state. CDK4i-induced senescence associated with loss of MDM2 is also observed in some breast cancer, lung cancer and glioma cell lines indicating that this is not limited to WD/DDLS cells in which MDM2 is overexpressed or in cells that contain wild type p53. MDM2 turnover depends on its E3 ligase activity and expression of ATRX. Interestingly, in seven patients the changes in MDM2 expression were correlated with outcome. These insights identify MDM2 and ATRX as new regulators controlling geroconversion, the process by which quiescent cells become senescent, and this insight may be exploited to improve the activity of CDK4i in cancer therapy.

## INTRODUCTION

Unlike other agents that target essential regulators of the cell cycle, PD0332991 (Palbociclib) has exhibited marked success in clinical trials [1]. PD0332991 specifically targets the CDK4 and CDK6 kinases [2, 3]. These kinases phosphorylate and inactivate the tumor suppressor Rb allowing cells to progress through G1-phase and commit to the cell cycle. CDK4 inhibition can induce Rb-positive cells to exit the cell cycle into a non-proliferative state, however, this can take the form of either quiescence, which is readily reversible, or senescence, which is more stable. In normal hematopoietic stem cells, CDK4 inactivation induces quiescence [4]. In tumor cells inhibiting CDK4 can induce senescence [5-7] or quiescence [8] depending on the cell type. Both outcomes are p53 and Ink4 independent, and what determines whether a cell undergoes quiescence or senescence is not clear.

Senescence is deemed a clinically favorable response to chemotherapy because of its stability and its capacity to stimulate tumor clearance by the immune system [9-11]. Senescence is characterized by gross changes in gene expression and chromatin, changes in cellular metabolism, and changes in the function of the cell. Senescence is typically a response to stress [12]. DNA damage, telomere attrition, or aberrant oncogene expression can induce p53, p16 and/or Arf leading to cell cycle exit and senescence [13-16]. However, senescence can also be triggered in a p53 and INK4 independent manner as well. [17-19].

*CDK4* deficiency in mice can limit tumor cell proliferation either directly by affecting Rb phosphorylation in the tumor cell, or indirectly by preventing the elaboration of a growth permissive tumor microenvironment [20-22]. In human clinical trials, CDK4 inhibitors (CDK4i) have had some success controlling tumor progression but why some patients respond well and others poorly is not understood [1, 23-25]. We hypothesized that the nature of arrest, vis a vis whether a cell undergoes quiescence or senescence, might contribute to the outcome. Thus, we set out to define the determinants distinguishing these outcomes.

Here we report that ATRX and MDM2 are both determinants of cellular outcome. Furthermore, in a small cohort of seven individual patients we were able to observe that MDM2 downregulation is associated with a positive response to CDK4i therapy auguring that a more detailed understanding of this pathway in the future may have substantial clinical impact.

## RESULTS

### CDK4 inhibition can induce senescence in a subset of Rb-positive liposarcoma cell lines

**Figure 1:**
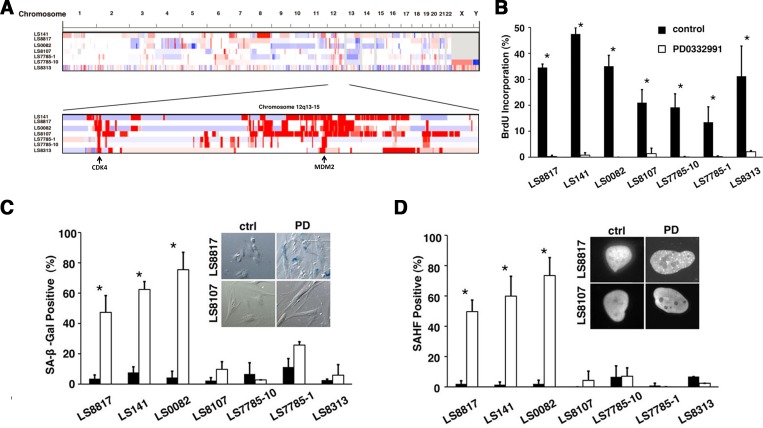
Inhibition of CDK4 triggers either senescence or quiescence in WD/DDLS (A) Copy number alterations in WD/DDLS cell lines. Amplification (red) and deletions (blue) were identified using the RAE algorithm [81]. (B) Cells were grown in the presence (white) or absence (black) of 1 μM PD0332991 for 2 days and labeled with BrdU for the last two hours before fixation and immunofluorescence. The percentage (mean and standard deviation) of cells that incorporated BrdU into the nuclear DNA was determined and plotted (*p<0.05). (C) Cells staining for SA-β-gal seven days after 1 μM PD0332991 treatment (white) or in untreated asynchronously growing cultures (black) were quantitated in three or more independent experiments and the mean and standard deviation plotted. (*p<0.05). Representative phase contrast micrographs for LS8817 and LS8107 are shown. (D) This panel is arranged as described in panel C but we determined the percentage of cells in which for HP1γ foci accumulated.

We looked at the response of a panel of seven Rb-positive patient derived WD/DDLS cell lines. These cell lines had common amplifications of *MDM2* and *CDK4* and a heterogenous assortment of copy number alterations as identified by array CGH (Figure 1A). As expected, within 48 hours PD0332991 induced the accumulation of G0/G1 cells in all the cell lines with significantly reduced phosphorylated Rb (Supplementary Figure 1). Why total Rb decreased in some cells but not others is not clear. Bromodeoxyuridine (BrdU) incorporation was also dramatically reduced in all the cells (Figure 1B). However, the accumulation of perinuclear senescence associated β-galactosidase (SA-β-gal, Figure 1C) and focal HP1γ, a marker of senescence associated heterochromatic foci (SAHF, Figure 1D), increased only in LS8817, LS141 and LS0082 cells. Similar results were seen at a range of doses as low as 100nM and as high as 10 μM. The failure of LS7785-1, LS7785-10, LS8107 and LS8313 to undergo senescence was not associated with increased apoptosis or adipocytic differentiation. Thus, we defined LS8817, LS141 and LS0082 cells as responders: cells that undergo senescence when treated with PD0332991. The other four cell lines were defined as non-responders, which undergo quiescence when treated with the drug.

Multiple markers are needed to characterize a cell as senescent [26]. Thus, we took some of these responders and non-responders and performed additional assays to examine other hallmarks of senescence. For example, senescence is a more stable form of growth arrest than quiescence. Consistent with this, after prolonged culture of the non-responder cells LS8107 and LS7785-1 in PD0332991 they incorporated BrdU within a day or two after removal of the CDK4i, whereas the responders LS8817 and LS0082 did not (Supplementary Figure 2). Further consistent with stable cell cycle exit clonogenic growth of LS8817 and LS0082 was significantly reduced three weeks after removal of CDK4i. In contrast, clonogenic growth of LS8107 was largely unaffected after removal of CDK4i (Supplementary Figure 2). LS141, LS8313, LS7785-1 and LS7785-10 were unable to grow at the low plating densities required for this assay.

Similar results were obtained using two other CDK4 inhibitors, LEE011 and LY2835219, in clonogenicity, SA-β-gal and SAHF assays. Additionally, reducing CDK4 with lentiviral transduced shRNAs in two of the responder cell lines (LS8817 and LS0082) and two of the non-responder cell lines (LS8107 and LS7785-1) gave similar effects. Representative data with one of the two hairpins is shown in Supplementary Figure 3. We did not perform this experiment in the other cell lines. Collectively, these results confirmed that the decision to exit the cell cycle into a senescent or quiescent state was not an off-target effect of PD0332991.

### Accumulation of unphosphorylated Rb has the same effect as CDK4 inhibition or knockdown

**Figure 2:**
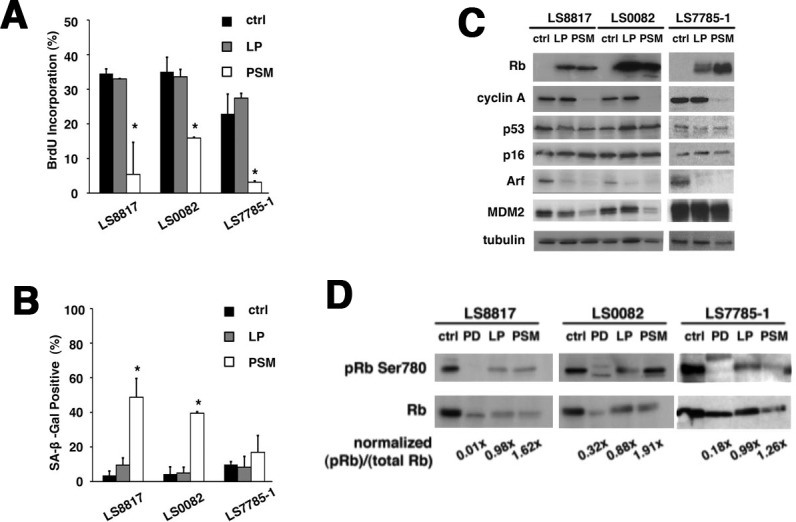
Accumulation of unphosphorylated Rb is sufficient to recapitulate the effect of CDK4 inhibition Cells were transduced with a lentivirus expressing either the large pocket of Rb (LP, grey) or a non-phosphorylatable large pocket of Rb (PSM, white) and selected for five days in blasticidin. The effect of these gene products on (A) BrdU incorporation, (B) SA-β-gal accumulation, and (C) protein expression is shown. (D) Endogenous phosphorylated Rb was measured by immunoblot. Extracts from cells treated with 1 μM PD0332991 (PD) and untreated control (ctrl) cells are shown for a comparison. These experiments were done at least three times with different pools of transductants with similar results each time. (*p<0.05).

Multiple CDK4 substrates have been identified and proposed to play roles in cell cycle exit [27, 28]. We wanted to determine if the accumulation of unphosphorylated Rb alone was sufficient to recapitulate the growth arrest phenotype of CDK4 inhibition or loss. To accomplish this we expressed the non-phosphorylatable large pocket mutant of Rb (PSM-Rb) [29] in two of the responder cell lines, LS8817 and LS0082, and one of the non-responder cell lines, LS7785-1. As a control we also expressed the wild type large pocket (LP), which could be inactivated by endogenous cyclin-dependent kinases. As expected BrdU incorporation was significantly curtailed by expression of PSM-Rb in all three cell lines, but not by LP (Figure 2A). SA-β-gal staining (Figure 2B) increased significantly only in the PSM-Rb expressing responder cell lines and not the non-responder. Arf and p16 were not increased and cyclin A was reduced in the senescent and growth arrested cells (Figure 2C). Phosphorylation of the endogenous Rb protein, while diminished relative to control cells was still detectable (Figure 2D), indicating that these growth arrested cells still had higher levels of CDK4 activity than the PD0332991 treated control cells. We did not perform this experiment in the other cell lines. Thus, the accumulation of unphosphorylated Rb, even in cells with active CDK4, could mimic the effect of CDK4i or CDK4 knockdown with respect to the choice of cells to become quiescent or senescent.

### Loss of MDM2 is associated with PD0332991-induced senescence in responder cells

**Figure 3:**
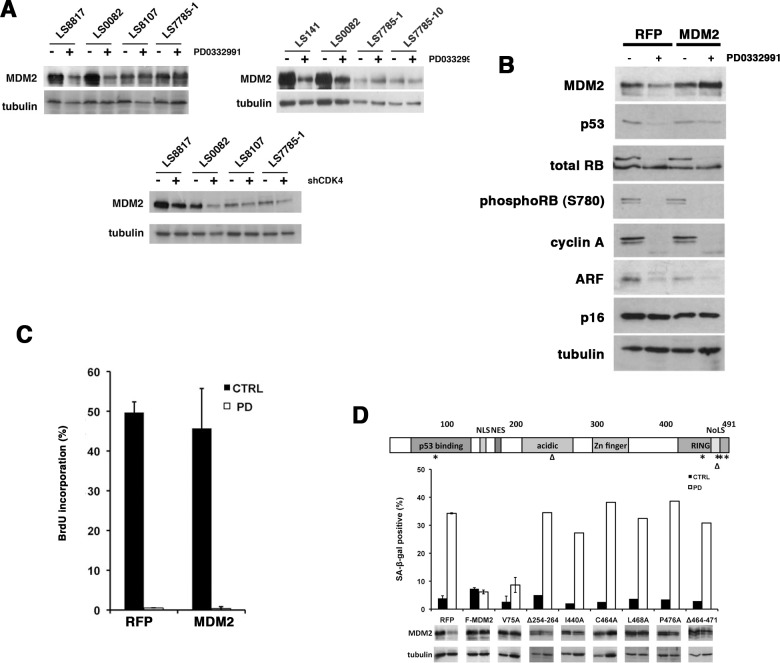
Enforcing MDM2 expression prevents senescence in WD/DDLS cells treated with PD0332991 (A) Top, Proteins were extracted from untreated cells or cells treated with 1 μM PD0332991 for two days and the expression of MDM2 determined by immunoblot. Bottom, Proteins were extracted from cells in which CDK4 levels were reduced with shRNA vectors. Cells transduced with vectors expressing a scrambled shRNA were used as a control. Tubulin is a loading control. This experiment was repeated at least three times and representative images are shown. (B) LS8817 cells were transduced with a lentivirus engineered to express MDM2 and selected for five days. Cells transduced with a lentivirus expressing RFP were used as a control. After five days of selection the remaining cells were treated with PD0332991 for two days and protein accumulation measured by immunoblot. (C) As described in the legend to panel B, the cells were labeled during the last two hours with BrdU prior to fixation. BrdU incorporation was measured as described in the legend to Figure 1B. (D) LS8817 cells were transduced with lentiviruses expressing the indicated mutants as described in the legend to panel B. Top, A diagram representing the domains of MDM2 and corresponding point mutations (*) or deletions (Δ) are indicated. Bottom, The accumulation of SA-β-gal was determined seven days after drug treatment as described in the legend to figure 1C. The expression of MDM2 was measured by immunoblot after 48 hours.

Because these cell lines, like WD/DDLS tumors, retain the characteristic amplification of chromosomal region 12q13-15 encoding the oncogenes *CDK4* and *MDM2* [30] we looked at whether the expression of MDM2 was different in the responder and non-responder cells. Responder cells expressed higher levels of MDM2 than non-responders (Figure 3A). However, it decreased approximately three-fold in the PD0332991 treated responder cells (LS8817, LS0082, LS141). On the other hand, it was relatively unchanged in the non-responders (LS8107, LS7785-1, LS7785-10, LS8313) (Figure 3A and data not shown). Furthermore, in responder cells, but not the non-responder cells, the level of MDM2 was reduced by CDK4 knockdown (Figure 3A). MDM2 levels were also decreased in the responder cells that underwent senescence induced by expression of PSM-Rb (Figure 2C), but not in the non-responder cell that underwent quiescence. This suggested that changes in MDM2 level, and not the absolute amount of MDM2, might be linked to the ability of CDK4 inhibitors to induce senescence.

### Enforced MDM2 expression can prevent PD0332991-induced senescence in responder cells

To assess whether continued expression of MDM2 would prevent senescence induced by PD0332991 treatment, we transduced the LS8817 cells with lentiviral vectors expressing a Flag-tagged MDM2 (F-MDM2) and treated them with PD0332991 after selection. As a negative control RFP was introduced. It is well known that enforced expression of MDM2 does not increase its abundance in cycling cells; however, enforced expression did prevent the PD0332991-induced reduction in MDM2 (Figure 3B). Accumulation of phospho-Rb and cyclin A were also reduced (Figure 3B). Enforcing MDM2 did not prevent growth arrest as evidenced by reduced BrdU incorporation (Figure 3C). However, it prevented the CDK4i-induced accumulation of SA-β-gal positive cells (Figure 3D). Thus, continued MDM2 expression was sufficient to prevent PD0332991 induced senescence; nevertheless, the cells exit the cell cycle.

### The E3 ligase activity of MDM2 is required to block senescence in PD0332991 treated cells

To identify which domains of MDM2 were necessary to suppress senescence we expressed different mutant alleles of *MDM2* and scored their effect in PD0332991 treated LS8817 cells. The V75A mutant disrupts binding to p53 [31]. The C464A mutant eliminates E3 ligase activity by disrupting the cross brace structure of the RING [32]. The I440A, L468A, or P476A mutants also eliminate E3 ligase activity but do so by selectively disrupting E2 binding [33]. The Δ254-264 mutant allele in the acidic domain disrupts multiple MDM2 protein interactions [34]. Of these only the V75A mutant was capable of suppressing the PD0332991-induced accumulation of SA-β-gal positive cells (Figure 3D). Another mutant that overlaps with the RING but also affects nucleolar localization (Δ464-471) could not suppress accumulation of SA-β-gal positive cells. This indicated that MDM2's ability to suppress PD0332991-induced senescence required its E3 ligase activity and the acidic domain but was independent of its ability to bind to p53.

### Knocking down MDM2 can induce senescence in non-responder cells

**Figure 4:**
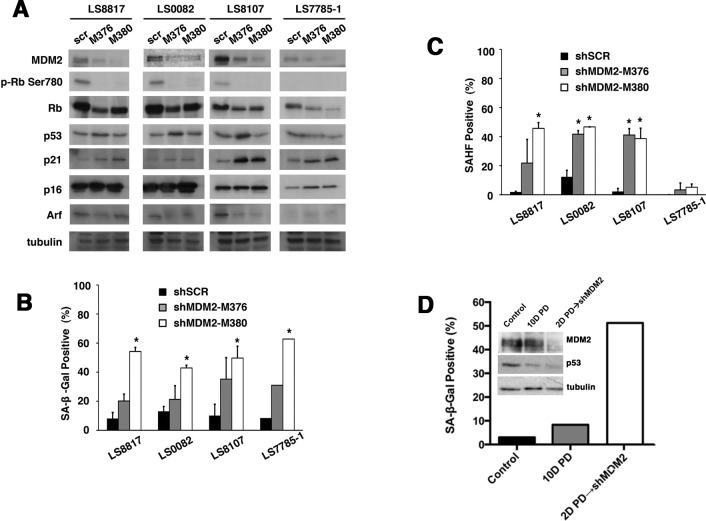
Loss of MDM2 can trigger senescence in WD/DDLS (A) The indicated cells were transduced with two different MDM2 knockdown lentiviral vectors (*M376* or *M380*) or a scrambled non-specific vector (*scr*) and selected in puromycin for five days prior to extraction of proteins for immunoblotting. (B) The percentage of cells staining for SA-β-gal 10 days after knockdown were quantitated as described in the legend to figure 1C. (*p<0.05). (C) This is arranged as in panel B but the percentage of cells staining for HP1γ foci were quantitated (*p<0.05). (D) LS8107 cells were first arrested in PD0332991 for 2 days and MDM2 was subsequently knocked down with *shM380* and cells selected as described in panel A. The effect on the accumulation of SA-β-gal positive cells and expression of MDM2 and p53 is shown. Tubulin is a loading control.

We then asked if knocking down MDM2 would be sufficient to induce senescence in the non-responder cells. To accomplish this, we generated two independent targeting vectors and transduced LS8107 and LS7785-1 non-responder cells with these or a scrambled control. Rb phosphorylation and the amount of cyclin A were reduced in the cells in which MDM2 was knocked down, indicative of growth arrest, even in the absence of PD0332991 (Figure 4A). Arf and p16 levels did not increase but there was a modest increase in p21, which is consistent with an increase in p53 activity when knocking down MDM2. p53 is wild type and functional in all of these WD/DDLS cells as demonstrated by the fact that nutlin-3 induced inhibition of MDM2-p53 interaction triggers apoptosis [35].

Nevertheless, reducing MDM2 by knockdown induced the accumulation of SA-β-gal positive cells (Figure 4B). SAHF also increased in LS8107 but not LS7785-1 (Figure 4C), which is consistent with the reports that not all senescent cells have the ability to form such foci [36, 37]. Likewise, knocking down MDM2 in LS8817 and LS0082 responder cells was sufficient in the absence of PD0332991 to induce growth arrest and accumulation of SA-β-gal and SAHF positive cells. The specificity of the shRNAs was confirmed because expression of a mismatched wobbled *MDM2* allele prevented MDM2 knockdown induced growth arrest and senescence. Thus, the senescence machinery was intact in non-responders, however, CDK4 inhibition induced cell cycle exit could not drive reduction of MDM2 in these cells.

CDK4 inhibition leads to a loss of p53 protein in WD/DDLS cell lines (Supplementary Figure 4) but MDM2 knockdown does not (Figure 4A). Thus, we wanted to ask if MDM2 knockdown could also induce senescence in a p53-independent manner. To accomplish this, we first treated non-responder LS8107 cells with PD0332991 to insure that they exited the cell cycle with reduced amounts of p53, and subsequently we infected these cells with a lentivirus vector expressing an shRNA to MDM2. In one experiment we removed PD0332991 two days after infection, and in the other we left the cells in PD0332991 for the duration of the experiment. The results were similar: p53 levels did not increase under either condition, and SA-β-gal positive cells accumulate after MDM2 knockdown (Figure 4D). Furthermore, knocking down p53 in LS8817 cells did not disrupt either CDK4 inhibitor induced down-regulation of MDM2 or accumulation of SA-β-gal (or SAHF, not shown), nor did it prevent MDM2 knockdown induced senescence (Supplementary Figure 5).

**Figure 5:**
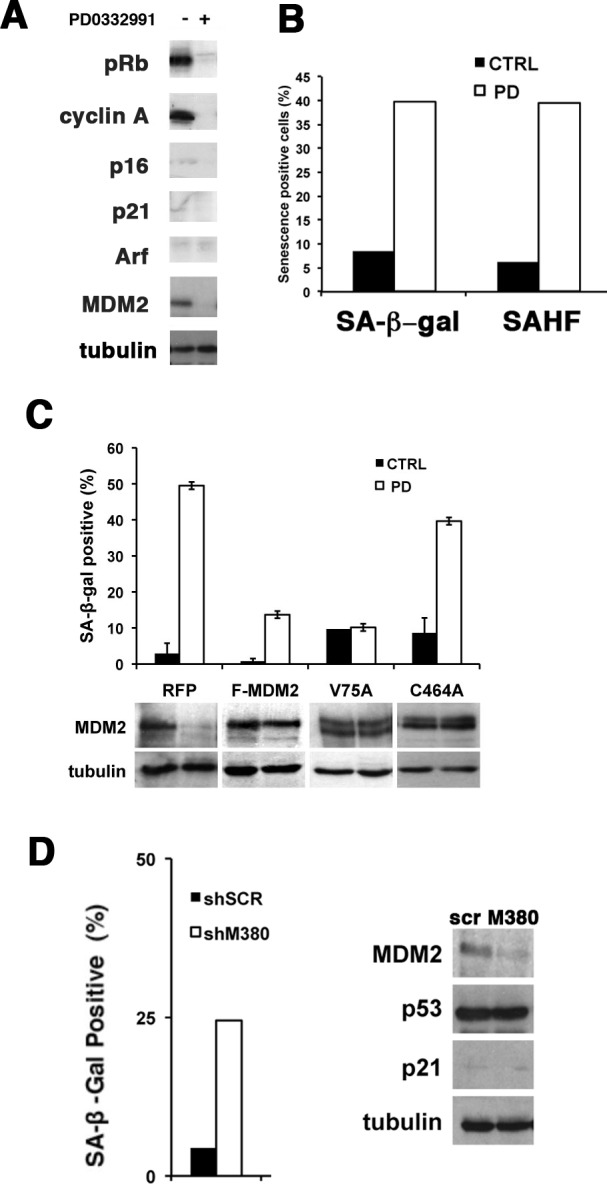
PD0332991 and MDM2 knockdown induce senescence is p53-mutant SNB19 cells (A) SNB19 glioma cell lines were treated with 1 μM PD0332991 and protein expression measured by immunoblot two days later. Tubulin is a loading control. (B) The accumulation of SA-β-gal and HP1γ foci was measured seven days after drug treatment as described in the legend to figures 1C and 1D. (C) Wild type and mutant MDM2 proteins were expressed in SNB19 glioma cells and analyzed as described in the legend to figure 3D. (D) MDM2 was knocked down in SNB19 cells with shM380 as described in the legend to figure 4A and the effect on accumulation of SA-β-gal positive cells (left) and p53 and p21 are shown (right).

We then performed another test of p53-independence. PD0332991 had already been shown to induce senescence in a collection of glioma cell lines [5] and one of these, SNB19, expresses a transcriptionally inactive mutant of p53 (R273H) that does not bind to DNA. We found that MDM2 levels decreased in these cells within 48 hours of drug treatment (Figure 5A) and confirmed that SA-β-gal and SAHF accumulated in seven days after treatment (Figure 5B). Clonogenic growth was also reduced after drug treatment (Supplementary Figure 2B). Similar to what we saw in WD/DDLS cells, enforced MDM2 expression did not affect PD0332991-induced cell cycle exit, but did prevent the accumulation of SA-β-gal (Figure 5C). This was E3 ligase dependent because mutation of the RING abrogated MDM2's ability to suppress senescence; but, it was unrelated to the ability of MDM2 to bind to p53 because mutation of the p53 binding domain did not affect its ability to prevent senescence (Figure 5C). Finally, knocking down MDM2 was able to induce accumulation of SA-β-gal positive cells (Figure 5D). Consequently, MDM2 loss can trigger a p53-independent senescence program.

### Amplification of MDM2 is not required for CDK4 inhibitor induced senescence

Because MDM2 is not overexpressed in SNB19 cells, it suggested that senescence mediated by MDM2 down regulation in response to CDK4i was not unique to those cells in which MDM2 is amplified. To examine this further we collected additional breast cancer (MDA-MB-453, T47D, ZR-75-1, and MCF7), lung cancer (A549, H1975, H358, H3122, Calu-1) and glioma (U87-MG, U251, and DBTRG-05MG) cell lines in which MDM2 was not amplified and treated these with PD0332991 to look at the hallmark characteristics of senescence. All underwent PD0332991-induced cell cycle exit within 24 hours, and MDM2 levels decreased in all except for H358, H3122 and Calu-1.

We looked further at some of the hallmarks of senescence in those cells in which we could assess clonogenicity following removal of PD0332991 (their p53 status is also indicated in the parentheses): T47D (L194F), H1975 (R273H), H3122 (WT), A549 (WT), and MCF7 (WT). Our results are summarized in table I. SA-β-gal accumulated in all the cells, except for H3122. HP1γ foci accumulated in all the cells except for H3122 and T47D. Nevertheless, the T47D and A549 cells returned to cycle upon drug removal whereas H3122 did not (Supplementary Figure 2B). Arrest was modestly more stable in MCF7 cells with approximately 40% of the cells returning to growth after the drug was removed. H1975 and H3122 were as strongly arrested as the LS8817, LS0082 and SNB19 cells with approximately 5% of the cells returning to cycle after drug was removed. Thus, we concluded that the amplification of MDM2 was not required for this response to CDK4i.

**Table I:** Effect of PD0332991 on hallmark characteristics of senescence

Cell line	p53 status	cell cycle exit	decrease in MDM2	accumulation of SA-β-gal	accumulation of HP1γ foci	clonogenic arrest
T47D	L194F	yes	yes	yes	no	no
H1975	R273H	yes	yes	yes	yes	strong
MCF7	wild type	yes	yes	yes	yes	moderate
A549	wild type	yes	yes	yes	yes	no
H3122	wild type	yes	no	no	no	strong

### PD0332991 triggers the dissociation of HAUSP from MDM2

**Figure 6:**
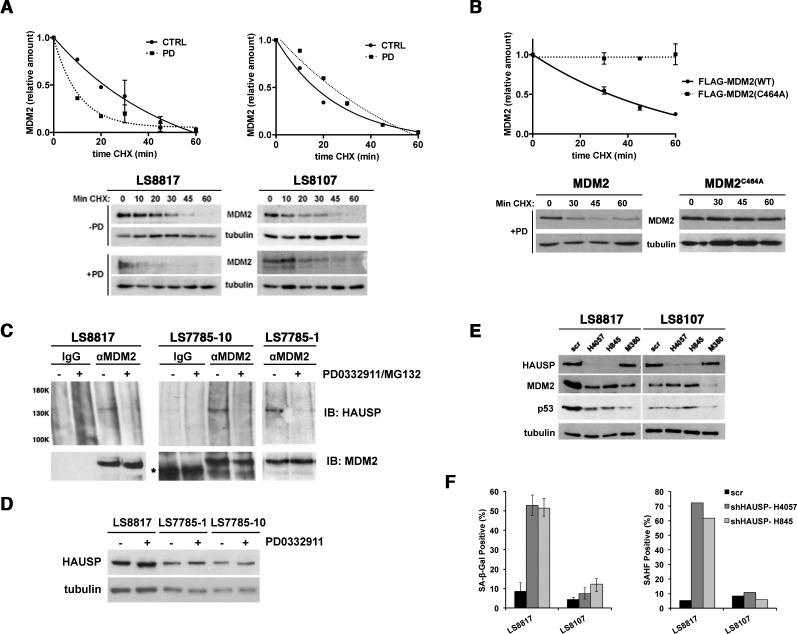
MDM2 is regulated post-translationally (A) LS8817 (left) and LS8107 (right) cells were treated with 1 μM PD0332991 for forty eight hours, and 75 μg/ml cycloheximide was added for the indicated times before proteins were extracted and amount of MDM2 measured by immunoblot. Tubulin is a loading control. Representative autoradiograms are shown below the graphs. (B) LS8817 cells were transduced with either a FLAG-tagged MDM2 or a FLAG-tagged C464A mutant of MDM2 as indicated and selected for five days. PD0332991 and cycloheximide were added as described in the legend to panel A. A representative immunoblot is shown and the graph was compiled from two independent experiments (mean + S.E.M.). (C) The indicated cell lines were treated with 1 μM PD0332991 for two days and 5μM MG132 added 2 hours prior to protein extraction. MDM2 was immunoprecipitated and HAUSP and MDM2 measured in the immunoprecipitate by immunoblot. IgG was used as a non-specific antibody control for the immunoprecipitation. (D) Proteins were extracted from asynchronously growing or cells treated with 1 μM PD0332991 for 2 days and the expression of HAUSP measured by immunoblot. Tubulin is a loading control. (E, F) The indicated cells were transduced with two different lentiviruses expressing shRNA targeting HAUSP or a scrambled control and selected for 10 days. All cells had exited the mitotic cycle. (E) Extracts were prepared and the accumulation of proteins detected by immunoblot. For comparison the effect of the MDM2 shM380 lentiviral knockdown is shown. (F) The accumulation of SA-β-gal and HP1γ positive cells was measured as described in other figures. This experiment was done twice with similar results.

We next wanted to gain some insight into the regulation of MDM2. In all the cell lines *MDM2* transcripts were modestly reduced by PD0332991, possibly reflecting the CDK4 inhibitor induced loss of p53 (Supplementary Figure 6). In contrast, MDM2 turnover was accelerated in LS8817, LS141 and LS0082 responder cells, but not in the LS8107, LS7785-1 and LS7785-10 non-responder cells (Figure 6A and Supplementary Figure 7). Adding the proteasome inhibitor MG132 to PD0332991-treated LS8817 or LS141 cells prevented the decrease in MDM2 (Supplementary Figure 7). Thus, the PD0332991-triggered loss of MDM2 was at least partially due to increased post-translational proteasome-dependent turnover. We did not look at turnover in LS8313 non-responder cells.

MDM2 turnover can be regulated by autoubiquitination and trans-ubiquitination by multiple E3 ligases. To determine if auto-ubiquitination contributed to MDM2 turnover after CDK4 inhibition we measured the turnover of *MDM2^C464A^* in LS8817 cells. This mutant can be ubiquitinated in trans but cannot be auto-ubiquitinated [38, 39]. As a control we expressed a Flag-tagged allele of *MDM2*. PD0332991 affected the turnover of wild type Flag-tagged MDM2 but not the C464A mutant (Figure 6B). Thus, PD0332991-enhanced turnover of MDM2 was dependent on auto-ubiquitination. Because E3 enzymes typically ubiquitinate both themselves and other substrates, it is not surprising that MDM2 E3 ligase activity would be necessary to suppress senescence and to induce its turnover in cells that would undergo senescence.

The interaction of MDM2 with the de-ubiquitinase HAUSP/USP7 is critical for inhibiting auto-ubiquitination induced turnover and driving the ligase activity towards other substrates [40, 41] prompting us to consider the possibility that the interaction with HAUSP might be different in responder and non-responder cells following CDK4 inhibition. However, it was not—the dissociation of HAUSP from MDM2 occurred in both LS8817 responder and LS7785-1 and LS7785-10 non-responder cells (Figure 6C). HAUSP levels did not change following PD0332991 treatment (Figure 6D). We did not look at this in the other cell lines. Thus, MDM2 must be stabilized after the dissociation of HAUSP in the non-responder cells. Consistent with this, knocking down HAUSP with two independent lentiviral hairpins in non-responder LS8107 and LS8313 cells does not reduce MDM2 levels (Figure 6E) nor increase the number of SA-β-gal or SAHF positive cells (Figure 6F). Nevertheless, these cells still undergo growth arrest. In contrast, reducing HAUSP in responder LS8817, LS141, or LS0082 cells was sufficient to induce arrest, reduce the level of MDM2 (Figure 6E), and induce the accumulation of SA-β-gal and SAHF positive cells (Figure 6F).

### ATRX is required for loss of MDM2 and for senescence induced by PD0332991

**Figure 7:**
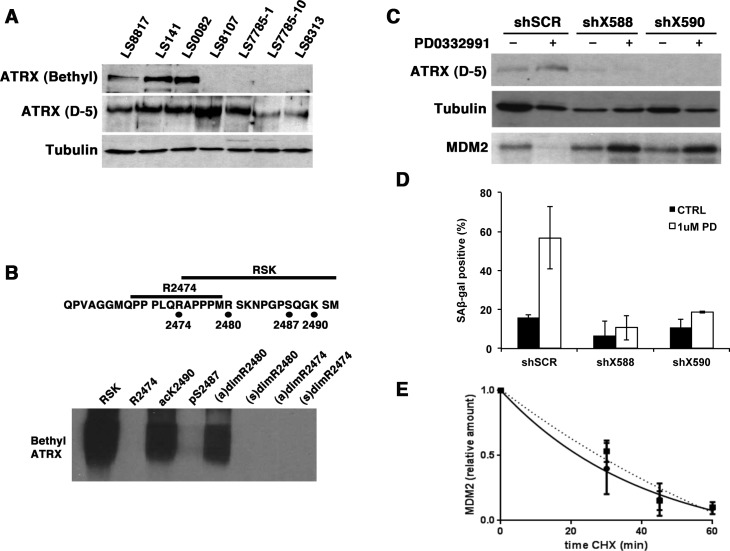
ATRX is needed for PD0332991 induced down-regulation of MDM2 and accumulation of SA-β-gal (A) Immunoblot. Extracts were prepared from asynchronously growing cells and blotted with the indicated antibodies. Tubulin was a loading control. (B) 1 ng of BSA-conjugated peptides were resolved on 8% SDS-polyacrylamide gels and after transfer to PVDF membranes their reactivity to the ATRX antibody from Bethyl Laboratories determined by immunoblot. Top, The 32 amino acid carboxyl-terminal sequence of ATRX protein is shown. Unmodified peptides are indicated with a bar and the modified residues are shown below with circles. The arginines were dimethylated in either the symmetric [(s)dim] or assymetric orientation [(a)dim]. Serine was phosphorylated [pS] and lysine was acetylated [acK]. (C-E). LS8817 cells were transduced with lentiviral vectors encoding shRNA targeting ATRX (shX588 or shX590) and a scrambled control (scr) and selected for five days. There was minimal effect on the proliferation of these cells for at least three weeks after transduction. (C) Protein expression was measured by immunoblot two days after drug treatment. (D) SA-β-gal accumulation was measured at seven days following treatment with PD0332991 as described in the legend to Figure 3D. (E) MDM2 turnover rates were measured in LS8817 shX590 expressing cells two days after treatment with PD0332991 and compared to turnover in untreated cells as described in the legend to figure 6A. This experiment was repeated twice and the mean and SEM are plotted.

Approximately one third of liposarcomas are characterized by ALT [42], a homologuos-recombination based mechanism to maintain telomeres in a telomerase-deficient cell. Thus we decided to look at whether ALT was associated with whether cells quiesce or senesce in response to CDK4 inhibitors. We measured ALT by both telomere-FISH and telomere restriction fragment assays [43, 44](Supplementary Figure 7). We also looked at the expression of ATRX as this protein is commonly not expressed in ALT-positive cells [44, 45]. All seven of the WD/DDLS cell lines expressed ATRX (Figure 7A) and ALT did not associate with response (Supplementary Figure 8). However, we noted that the ATRX antibody from Bethyl Laboratories could not detect ATRX in all four of the non-responder cells. Thus, expression of this isoform of ATRX was associated with response.

The antibody from Bethyl Laboratories is raised to an epitope in the last 50 amino acids of ATRX. Four modifications are reported in this region (phosphosite.org). To determine which, if any of these, block the binding of the Bethyl antibody we generated four modified peptides and two unmodified control peptides and coupled them to BSA. Conjugation was confirmed by HPLC. We resolved these by SDS-PAGE and after transfer to PVDF membranes we blotted with the Bethyl antibody. The peptide encoding arginine 2474 was outside the epitope recognized by this antibody. The antibody could recognize the other peptide that contained arginine 2480, serine 2487 and lysine 2490. Phosphorylation of serine 2487 or symmetric dimethylation of arginine 2480 interfered with antibody binding. Asymmetric dimethylation of arginine 2480 and acetylation of lysine at 2490 could not (Figure 7B). Serine 2487 phosphorylation and unmodified lysine 2490 were confirmed in ATRX immunoprecipitates from LS8107 analyzed by MALDI-TOF mass spectroscopy; however, we were unable to detect peptides containing the arginine 2480 residue. Thus, we suspect that C-terminal modifications of ATRX may affect its activity in CDK4 inhiibitor induced senescence but not its role in telomere maintenance.

Consequently, we asked whether ATRX was needed for CDK4i to induce senescence. To explore this, we acutely knocked down ATRX in LS8817 cells with two different shRNA vectors (Figure 7C) and measured the effect on accumulation of SA-β-gal positive cells. In these ATRX-deficient cells, the drug still induced cell cycle exit, but did not induce accumulation of SA-β-gal positive cells (Figure 7D). MDM2 did not decrease (Figure 7C), nor was its turnover accelerated (Figure 7E). Similar results were seen in LS0082 and SNB19 glioma cells—knocking down ATRX prevented CDK4 inhibitor induced senescence and MDM2 levels did not decrease. This indicated that ATRX was required for the regulation of MDM2 in responder cells treated with CDK4 inhibitors, and suggests that this might be prevented by C-terminal modifications.

### MDM2 loss associates with clinically favorable responses to PD0332991

How to define a senescent cell is problematic because not all hallmarks of senescence are seen with all inducers and many hallmarks are not unique for senescence [26]. Nevertheless, we wanted to determine if ATRX and MDM2 turnover were important, particularly in respect to the usefulness of CDK4 inhibitors in the clinic. Because the ATRX antibody from Bethyl Laboratories is unable to be used for IHC we could not examine this; however, we were able to look at MDM2 levels in seven paired pre- and post-treatment biopsies from individual WD/DDLS patients enrolled in our phase II clinical trial with PD0332991 (NCT01209598) [24].

**Figure 8:**
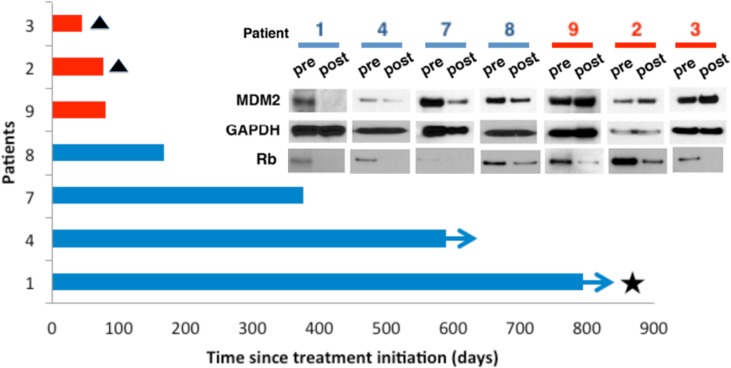
MDM2 loss is associated with patient response to Palbociclib Seven patients with measurable advanced WD/DDLS confirmed positive for CDK4 amplification and Rb protein expression, and whom had progressed on at least one systemic therapy prior to enrollment (clinicaltrials.gov identifier NCT01209598), consented to pre-treatment biopsies taken prior to therapy and post-treatment biopsies taken after receiving one dose of oral PD0332991 (125mg daily for 21 days). Tumor response was assessed with CT scan by a reference radiologist every 6 weeks for 36 weeks, and every 12 weeks thereafter. Patients were followed up until March 2014. Three patients (red bars) did not derive clinical benefit and stopped treatment within 84 days. Two of these patients (#2 and #3, triangles) died on study and are indicated with a triangle. The third patient, #8, came off study as their disease progressed. Four patients (blue bars) had demonstrable clinical benefit, remaining on treatment for more than 84 days. Two of these progressed after 168 (#8) and 376 (#7) days respectively, while two remained on treatment with ongoing benefit (arrows), one achieving a complete response (star). (Inset) Extracts were prepared from pre- and post-treatment biopsies of protein expression measured by immunoblot. GAPDH was a loading control.

The specifics of the trial, treatment regimen, and timing of biopsy as well as the criteria for patient outcome are described in the methods. Three of these patients had stable disease for more than 1 year, one had a complete remission by RECIST 1.1 criteria, and three did poorly on the drug. To measure MDM2 we extracted protein from pre- and post-treatment biopsies and performed immunoblots. We also blotted GAPDH as a loading control and phosphorylated Rb to confirm that the drug had hit the target. MDM2 was reduced in all four post-treatment samples from patients who performed well, and was not reduced, and even increased in patients who performed poorly (Figure 8). phospho-Rb diminished in all the PD0332991-treated tissues relative to the pre-treatment biopsy. Because of the limited amount of material we could not look at ATRX reactivity with the Bethyl antibody. Thus, PD0332991-induced down regulation of MDM2 associates with a positive response to therapy.

## DISCUSSION

Cellular senescence is a stable non-proliferative state in which cells typicaly accumulate characteristic hallmarks such as SA-β-gal and SAHF, and elaborate a senescence associated secretory program. Multiple hallmarks are collectively used to define this state, but the stability of the proliferative arrest is its key distinguishing feature [26, 46]. Interest in senescence has risen because it is a cell-autonomous barrier to oncogenic transformation [14, 15, 47, 48], and because the activation of a senescent associated secretory program may recruit immune modulators that ultimately affect tumor killing [9, 49].

In untransformed cells, senescence can be triggered by a variety of stresses, such as the accumulation of reactive oxygen species, DNA damage, aberrant oncogene expression, or telomere attrition. These signals induce cell cycle exit and senescence in a p53-dependent manner [12]. CDK4 inhibitors also induce cell cycle exit in untransformed cells, but typically these cells do not senesce [4]. Nevertheless, CDK4 inhibitors induce senescence in some, but not all, transformed cells [5-8]. This is independent of p53 and Ink4 [7]. Why some WD/DDLS cells undergo senescence and others do not following CDK4 inhibition was not clear. We were unable to link this to the activity of SCF^skp2^, the accumulation of p21 or E2F7, the expression of p300 or twist, or the accumulation of DNA damage, telomere attrition, or production of ROS, all well established inducers of senescence whether p53 dependent or independent [17-19, 50]. Because senescence is a preferred outcome of cell cycle exit induced by chemotherapy, and CDK4 inhibitors have achieved Breakthrough Therapy Designation from the FDA and have had some success in treating patients with WD/DDLS, it seemed important to understand this.

Geroconversion describes the transition of a cell from quiescence to senescence [46, 51]. Blagosklonny's group showed that mTOR activity could affect the outcome of p53 dependent arrest [46, 51, 52]. Here we report that the regulation of MDM2 and the expression of ATRX can also affect whether transformed cells undergo quiescence or senescence, particularly in response to CDK4 inhibition. Furthermore, we were able to correlate changes in MDM2 with patient outcome because seven patients enrolled in our palbociclib clinical trial (NCT01209598) consented to providing pre- and post-treatment biopsies, suggesting that geroconversion might account for the clinical activity of this class of drugs. Thus we accomplished what we set out to do—identify determinants that can distinguish whether CDK4 inhibition will induce quiescence or senescence, and in so doing we have unexpectedly identified two new molecular players (ATRX and MDM2) and an event (MDM2 turnover) regulating geroconversion.

**Figure 9:**
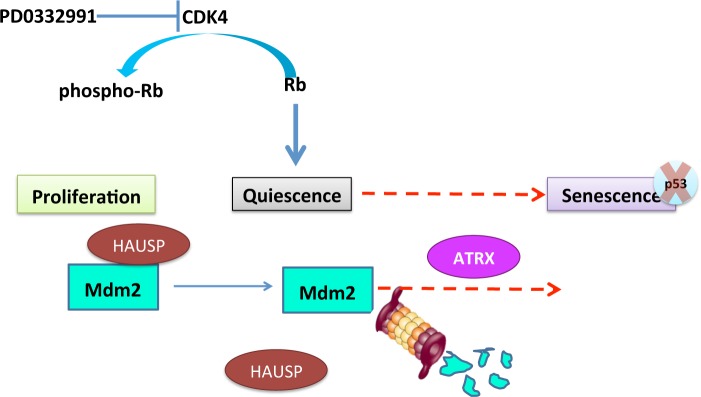
Summary As described in the text, PD0332991 can induce cell cycle exit accompanied by the dissociation of HAUSP from MDM2. Geroconversion, or the transition of cells to senescence, indicated by the red arrow, is dependent on proteosomal degradation of MDM2 and expression of ATRX. p53-independent mechanisms can contribute to senescence induced by CDK4 inhibition and MDM2 knockdown.

As summarized in Figure 9, our data indicate that geroconversion depends on the accelerated rate of MDM2 turnover following the dissociation of the deubiquitinase HAUSP from MDM2. CDK4 inhibition induces cell cycle exit and this primes cells for MDM2 auto-ubiquitination, however, something in non-responder cells is preventing this. Intriguingly, expression of the chromatin-remodeling enzyme ATRX is also important for geroconversion. Because ATRX deficiency prevents the loss of MDM2 we hypothesize that ATRX directly regulates a program that impinges on MDM2 regulation. Furthermore, because the phosphorylation of ATRX at serine 2487 is associated with the inability of cells to undergo geroconversion following CDK4 inhibition, we suspect that this modification affects this activity. More detailed mechanistic studies can examine each part of this model in the future as discussed below.

### MDM2 turnover and the role of ATRX in geroconversion

In cells that fail to undergo geroconversion something is stabilizing MDM2 following dissociation of HAUSP. The dephosphorylation of serine 395 by the WIP1 phosphatase and the binding of MDM2 to MDMX or ENIGMA/PDLIM7 had been reported to stabilize MDM2 [53-55]. However, neither the phosphorylation state of serine 395 or the interaction of MDM2 with MDMX or ENIGMA/PDLIM7 could segregate the seven cell lines based on their class-specific responses. While it is formally possible that each non-responder cell line evolves a different mechanism, we do not favor this hypothesis because we could see that the C-terminal modification of ATRX is class-specific. Accumulation of the serine 2487 phosphorylated ATRX protein is detected in all four non-responder cell lines, at least when judged by immunoblot reactivity.

How might ATRX regulate MDM2 turnover? ATRX binds to repeats in telomeric, subtelomeric, and pericentric regions and at repeat motifs that affect gene expression [56-60]. Like other SWI/SNF chromatin regulators, ATRX directly binds and slides, remodels, or removes histones from nucleosome complexes [61]. Together with DAXX, ATRX promotes the integration of H3.3 into histone monomers [58, 62-64]. ATRX can also bind to chromatin by way of direct interactions with heterochromatin protein 1 (HP1) and through combinatorial readouts of H3K9me3 and H3K4me0 histone monomers. ATRX can regulate expression of specific genes: for example, ATRX binding prevents the accumulation of macroH2A at the alpha-globin locus thus promoting gene expression. We suspect that ATRX modulates a gene expression program, the products of which impinge upon MDM2 regulation, and phosphorylation of the carboxyl terminus prevents this. Alternatively, it remains possible that ATRX indirectly effects MDM2 regulation.

### What is the substrate for MDM2?

Our data demonstrate that the loss of MDM2 accompanies and drives genroconversion. The E3 ligase activity of MDM2 is required to prevent geroconversion in cells treated with CDK4 inhibitors. Because knocking down MDM2 is sufficient to induce senescence in non-responder cells our data suggests that MDM2 is directly ubiquitinating a senescence-activating protein (SAP). Identifying this SAP(s) is important.

While we initially expected that MDM2's ability to regulate p53 would account for its activity in geroconversion, it clearly did not. First, knocking down p53 does not affect either CDK4 inhibitor induced senescence or senescence induced by MDM2 knockdown in WD/DDLS cell lines. Breast, glioma, and lung cancer cell lines expressing p53 mutant proteins can senesce when treated with CDK4 inhibitors or following MDM2 knockdown as well. Finally, senescence can be prevented by enforced expression of an MDM2 mutant that fails to bind p53. Additionally, if p53 does accumulate, such as when WD/DDLS cell lines are treated with nutlin-3a, cells undergo apoptosis [35, 65]. Collectively, this indicates that this MDM2 regulated pathway is clearly p53-independent. We do not understand how p53 levels and activity are controlled by CDK4 inhibitors.

The MDM2 interactome is a place to look for a potential SAP. There are a few hundred MDM2 interacting proteins [66]. Approximately 45 of these can be considered high confidence interacting proteins because they are either in the same KEGG functional group and/or multiple groups reported the interaction using different assays [67]. Several of these are implicated in senescence, including MDM4, UBC, RB, YY1, HDAC1, DAXX, and PML and Sp100 [54, 68-77]. However, most of these modulate senescence through a p53 pathway lessening their likely impact in CDK4 inhibitor induced senescence; nevertheless, a p53-independent pathway might have been overlooked. Alternatively, there are other geroconversion specific substrates to be identified.

### Geroconversion: a new target for CDK4 inhibitor chemotherapy

Our data is consistent with an association of favorable clinical outcome with CDK4 inhibitors and the drug-induced down-regulation of MDM2 in WD/DDLS. We hypothesize that geroconversion underlies the efficacy of CDK4 inhibitors in the clinic. Although CDK4 inhibitors are somewhat effective alone in WD/DDLS [24, 25], we suspect that their activity can be improved in combination with other agents; however, what agents to select is not obvious.

In breast cancer and breast cancer cell lines, CDK4 inhibitors have been used in combination with estrogen receptor antagonists and AKT inhibitors and reported to enhance their clinical or cytostatic activity, respectively. Other studies in pancreatic cancer also favor combinations with PI3-kinase and mTOR inhibitors [78]. Mechanistically, it is thought that CDK4 inhibition limits cell proliferation after the initial signaling blockade decays and cyclin D1 levels begin to rise [79, 80]. However, our data suggests an alternative interpretation. We suggest that CDK4 inhibitors alone establish a strong G1 arrest in those cells, but geroconversion is less efficient, and perhaps even incomplete. The other drugs alter the signaling milieu creating a more favorable environment for geroconversion. Consistent with this it is worth noting that the breast cancer cell line, MCF7, is strongly arrested with CDK4 inhibitors, MDM2 levels are decreased and there is some conversion to senescence as measured by multiple hallmarks; however upon removal of the CDK4 inhibitor half the cells can return to cycle. We suspect that combining this with AKT inhibitors will alter the mTOR related signaling environment, which is known to affect geroconversion, and ultimately stabilize the genetic changes required to enforce MDM2 turnover. Intriguingly, in LS8817 both CDK4 inhibitors and expression of PSM-Rb are able to induce geroconversion complete with stable reduction in MDM2; however serum starvation could not induce geroconversion or loss of MDM2. We interpret this to suggest that receptor tyrosine kinase or nutrient signaling can impact on this transition once cells have exited the cell cycle. Future studies using well defined cellular models in which geroconversion can be studied as cells progress from quiescence to senescence will allow us to address this directly, and clarify some of the confusion that has arose by studying cell cycle exit AND senescence at the same time.

## METHODS

### Cell culture

Cell lines were developed from WD/DDLS tumors resected from surgical patients after obtaining informed consent. LS8817 and LS0082 have previously been described using the nomenclature DDLS8817 and WD0082. DNA was extracted from cell lines using standard protocols (QIAGEN DNEasy) and lineage confirmed by copy number array to confirm amplification of segment 12q13-15 (Agilent 244K according to manufacturer's specifications). Analysis of comparative genomic hybridization data was performed using a custom pipeline, which conducts the standard circular binary segmentation from the R/bioconductor DNAcopy library and processes all samples with the RAE algorithm [81].

Cell lines were maintained in DME HG supplemented with 10% heat-inactivated fetal bovine serum and 2mM L-glutamine.

### Gene expression analysis

RNA was extracted from cells (RNEasy, QIAGEN) and reverse transcription performed after treatment for 7 days with PD0332991 (Selleckchem).

### Gene targeting by shRNA

shRNA were delivered in the pLKO.1 vector (Open Biosystems) and infected cells selected using puromycin (1μg/ml); infection with a virus carrying a scramble control (CAACAAGATGAAGAGCACCAA) was used as a control in all experiments utilizing shRNA. Cell lines were treated with PD0339221 or shRNA directed against CDK4 (GAGATTACTTTGCTGCCTTAA), MDM2 (M376, TTCACTATTCCACTACCAAAG; M380, TACTAGAAGTTGATGGCTGAG), CDK6 (GACCTGGAAAGGTGCAAAGAA), or ATRX (588, GCCTGCTAAATTCTCCACATT; 590, CGACAGAAACTAACCCTGTAA) for 48 hours to 7 days.

To rescue the MDM2 knockdown we infected cells with a lentivirus (pLOC, Open Biosystems) encoding either an *MDM2* expression cassette containing a mismatched sequence (ACTATTCTCAACCCTCAACTTCTA) or an *RFP* cassette. 24 hours later after transduction, positive cells were selected in media containing 3μg/ml blasticidin and selection was maintained throughout the experiment. Five days after blasticidin selection began we transduced the cells with a second lentiviral vector encoding either the *shM380* sequence targeting MDM2 or a scrambled sequence (*shSCR*) as described above. 24 hours later these cells were selected in media containing both blasticidin and 3μg/ml puromycin.

### Antibodies

Antibodies against CDK4 (3F121), MDM2 (SMP14), total Rb (IF8), cyclin A (H432), p16 (C20), p53 (DO-1 and Bp53-12), tubulin (C20) and FLAG (M2) were obtained from Santa Cruz Biotechnology, phospho-Rb 780 (#9307) from Cell Signalling, Arf (3642) from Abcam, and ATRX (A301-045A) from Bethyl Laboratories. Treated cells were lysed with buffer composed of 50mM Tris-HCl, pH7.4, 250mM NaCl, 5mM EDTA, 0.5% NP40, 2mM PMSF, and supplemented with protease inhibitors. Forty to eighty micrograms of protein were resolved by SDS-PAGE and transferred to PVDF membranes. Membranes were incubated overnight with antibodies.

Extracts were prepared from pre-treatment biopsies within two weeks before the first dose of the drug, and post-treatment biopsies were collected within six days of the start of the second cycle. Extracts were prepared in 50mM Tris-HCl, pH7.4, 150mM NaCl, 1mM EDTA, 1% NP40, 0.25% sodium deoxycholate and supplemented with mini-protease inhibitor cocktail (Roche). Tumor response was assessed by reference radiologist by CT scan every six weeks for 36 weeks, and every 12 weeks thereafter. The clinical trial was approved by the Institutional Review Board of Memorial Sloan-Kettering Cancer Center and all patients provided written informed consent (NCT01209598).

### Senescence analyses

Cells were plated at a concentration of 25,000 per well in a 4-well chamber slides (Lab-Tek) and treated for seven days with drug and stained for senescence-associated β-galactosidase (Cell Signaling kit #9860). Cell number was quantitated by DAPI staining and β-galactosidase staining quantitated as a proportion of total cells.

Senescence associated heterochromatic foci were quantitated after cells were fixed with 4% paraformaldehyde, permeabilized with 0.1% Triton, blocked with 2% FBS, and stained with antibodies against HP1γ (1:5000 dilution, 2MOD-1G6 Millipore). Senescent cells were identified by immunofluorescence after treatment of slides with anti-mouse secondary antibodies and quantitation of focal SAHF as a percent of total cells (Leica Upright Confocal SP5 confocal microscope).

### Clonogenic growth assays

Cells were treated with 1 μM PD0332991 for seven days, collected and then 1000 cells were plated and cultured for three weeks in drug-free medium before staining with crystal violet.

## SUPPLEMENTARY MATERIAL FIGURES


