# Supplementary material for: MDM2 turnover and expression of ATRX determine the choice between quiescence and senescence in response to CDK4 inhibition
Source: Oncotarget. 2015 Jan 31;6(10):8226–43. doi: 10.18632/oncotarget.3364 (PMC4480747; doi:10.18632/oncotarget.3364)
Supplement: Supplementary file 1 [file oncotarget-06-8226-s001.pdf]

MDM2 turnover and expression of ATRX determine the choice between quiescence and senescence in response to CDK4 inhibition

Supplementary Material

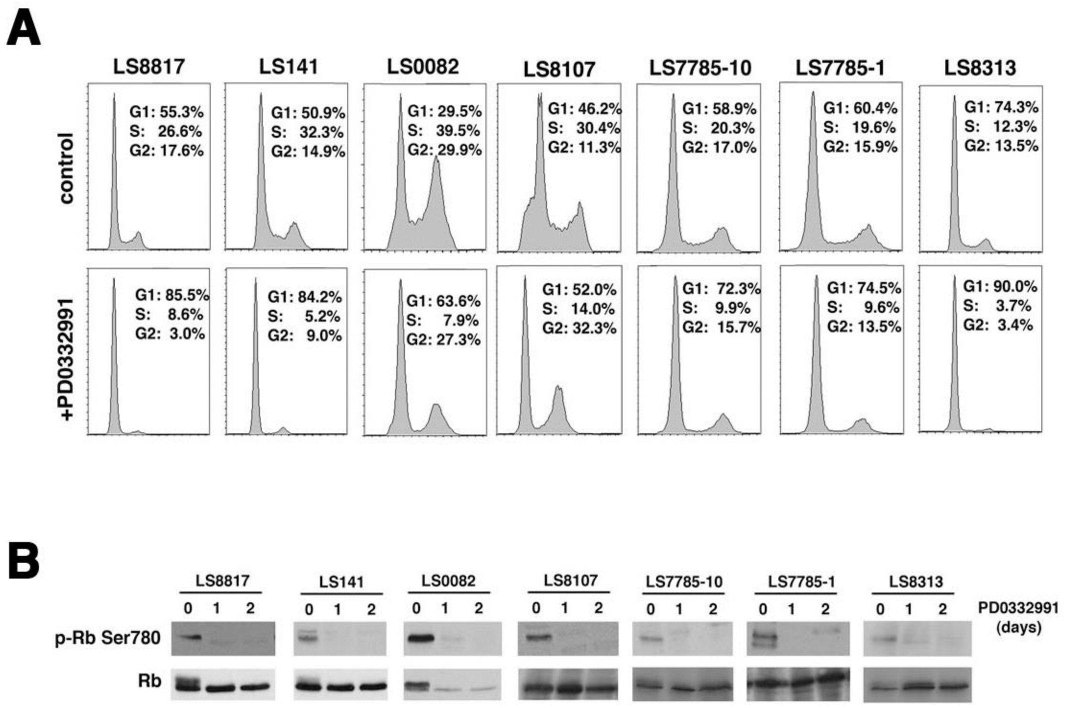

Supplementary Figure 1: PD0332991 can induce either quiescence or senescence in Rb-positive liposarcoma cell lines. (A) Cells grown in the presence of 1 μM PD0332991 for two days were stained with propidium iodide and analyzed by flow cytometry to determine their distribution in the cell cycle. The inset numbers are the means of three independent experiments. Untreated asynchronously growing cells (control) are included for comparison. (B) The amount of serine 780 phosphorylated and total Rb were detected by immunoblot in extracts from asynchronously growing cells (0) and cells treated with 1 μM PD0332991 for either 1 or 2 days (representative experiment; n≥3 for each cell line).

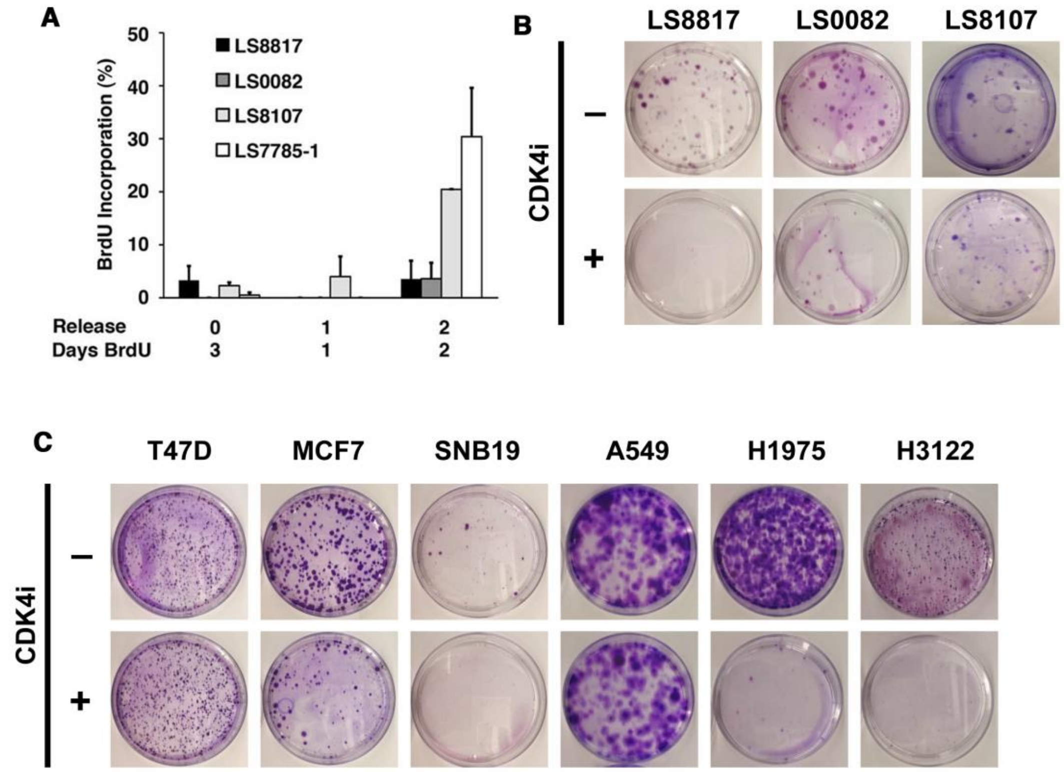

Supplementary Figure 2: CDK4 inhibitors can induce long term stable growth arrest after drug removal in some cell lines. (A) The four WD/DDLS cell lines indicated were treated with 1 μM PD0332991 for seven days and released into drug-free medium containing BrdU for the number of days indicated below each graph. Error bars represent the standard deviation of the mean calculated from three independent experiments. (B) Representative images of clonogenic growth of WD/DDLS cells treated with PD0332991 (PD) for seven days are shown. After seven days the cells are collected and replated at low density and the number of colonies measured with crystal violet staining 3 weeks later. Similar results were obtained at doses as low as 250nM and using LEE011 or LY2835219. (C) Same as in panel B but showing representative images of clonogenic growth in breast cancer (T47D, MCF7), glioma (SNB19), and lung cancer (A549, H1975 and H3122) cell lines.

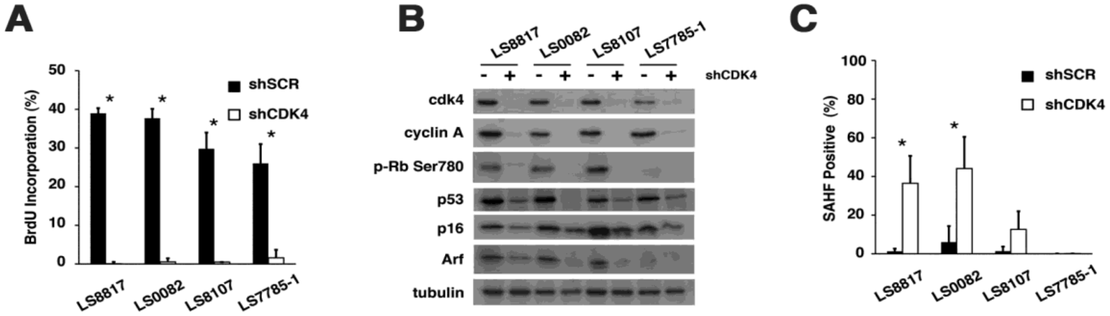

Supplementary Figure 3: Knocking down CDK4 induces growth arrest and senescence. Cells were selected in puromycin for five days following lentiviral infection with an shRNA targeting CDK4 or a scramble control shRNA and proliferation was measured by BrdU incorporation (A), expression of protein by immunoblot (B), and the number of cells with HP1γ foci by immunofluorescence (C). These results were quantitated in three or more independent experiments for each cell line and representative data is shown (*p<0.05).

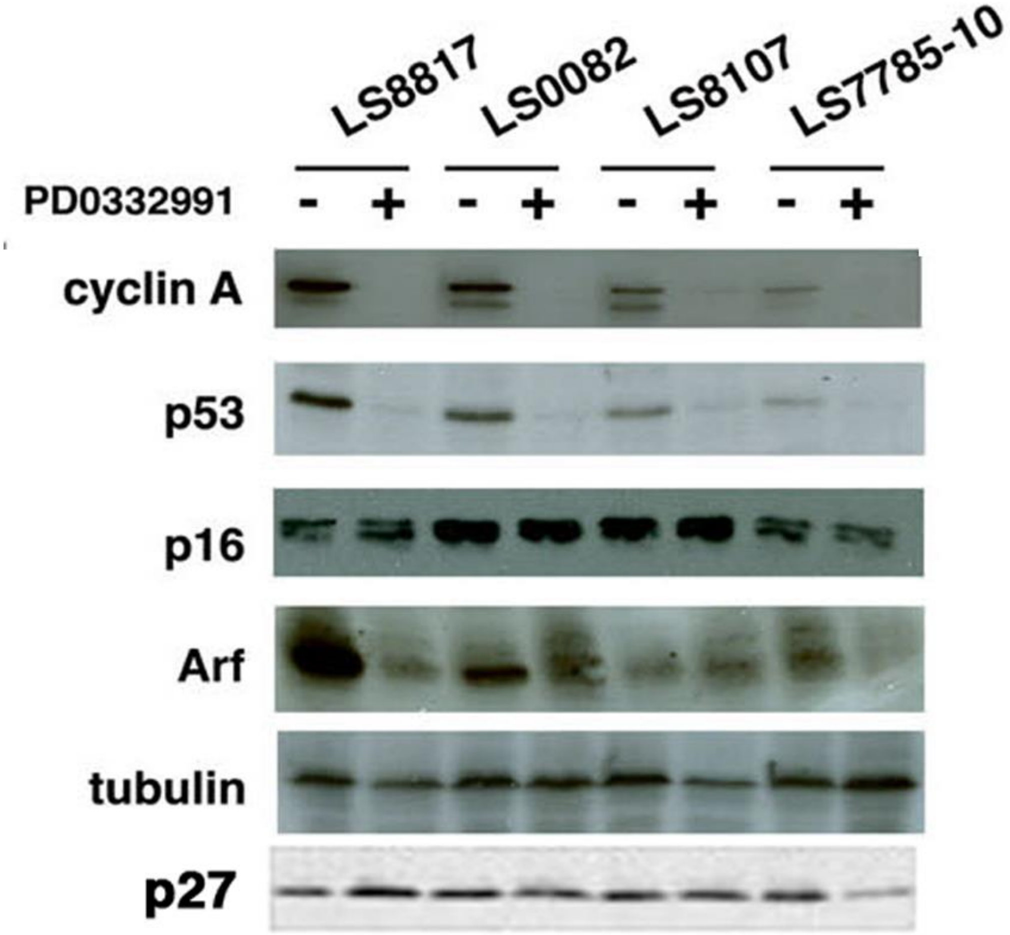

Supplementary Figure 4: p53 levels are reduced in WD/DDLS cells treated with PD0332991. The cells were treated with the drug for 2 days prior to protein extraction and the level of proteins assessed by immunoblot. Tubulin is a loading control. Loss of cyclin A protein confirms the drug induced cell cycle exit.

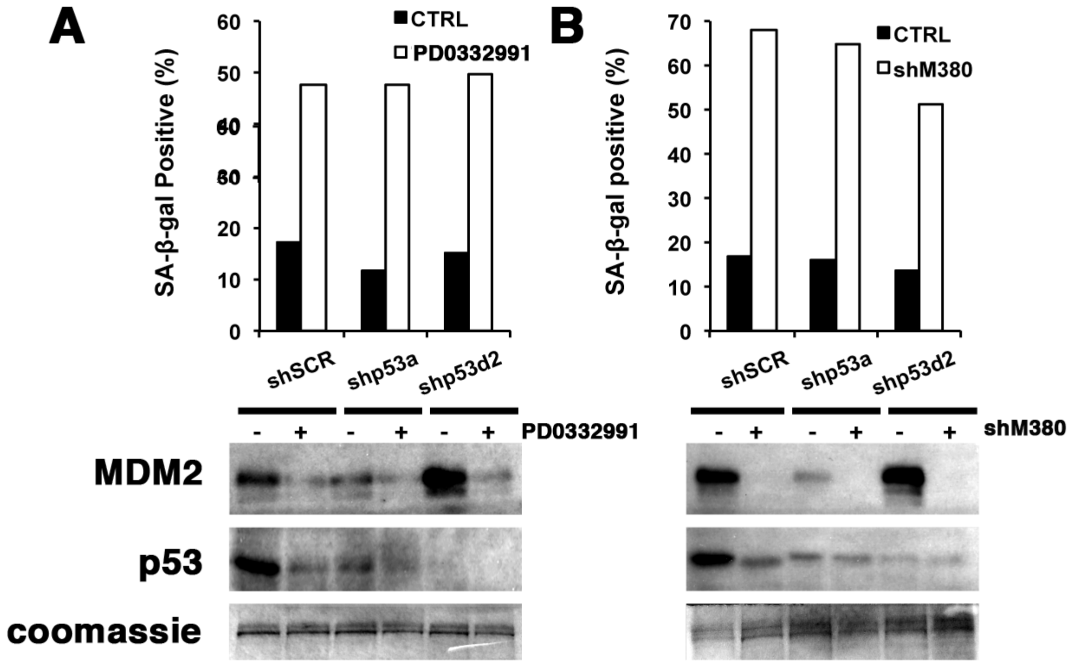

Supplementary Figure 5: p53 is not required for PD0332991 or MDM2 knockdown induced senescence in LS8817 cells. LS8817 cells were infected with lentiviruses expressing either a scrambled (shSCR) or two independent p53 targeting (shp53a and shp53d2) shRNAs. After selection these cells were either treated with PD0332991 (A) or MDM2 was reduced by superinfection with a lentivirus expressing shM380 (B). The accumulation of senescence associated β-galactosidase (top), and the expression of MDM2 and p53 were determined by immunoblot (bottom). Coomassie staining of the gel indicated equivalent loading.

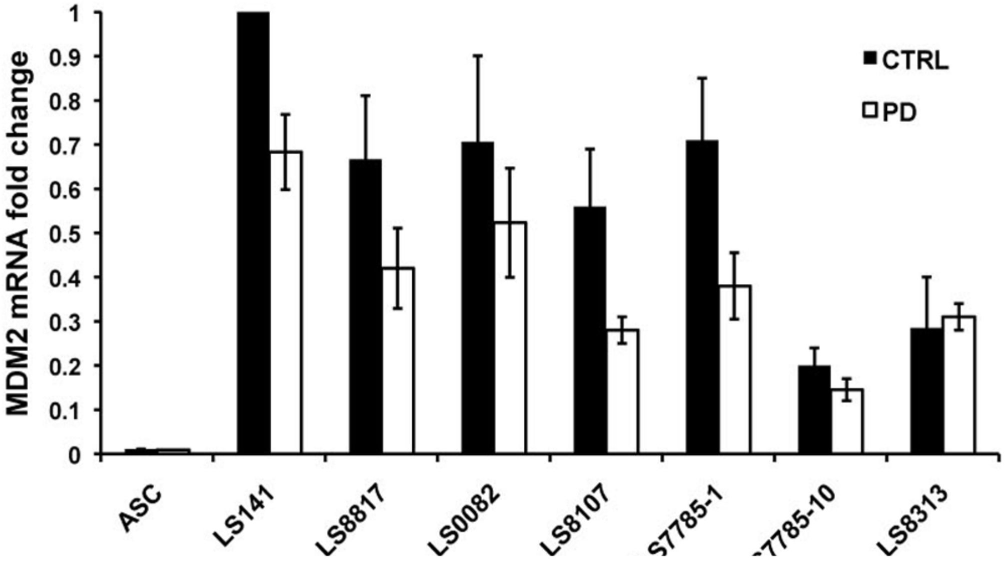

Supplementary Figure 6: MDM2 transcripts are similarly reduced in all the WD/DDLS cells after treatment with PD0332991 regardless of the outcome of this arrest. The cells were treated with 1 μM PD0332991 for two days and the effect on MDM2 transcript levels determined by qPCR. This experiment was repeated at least three times on different biologic replicates. All the amounts of mRNA detected were normalized to the amount observed in control untreated LS141 samples, which was set to 1. The level of transcript in a primary adipocyte stem cell (ASC) is shown for comparison.

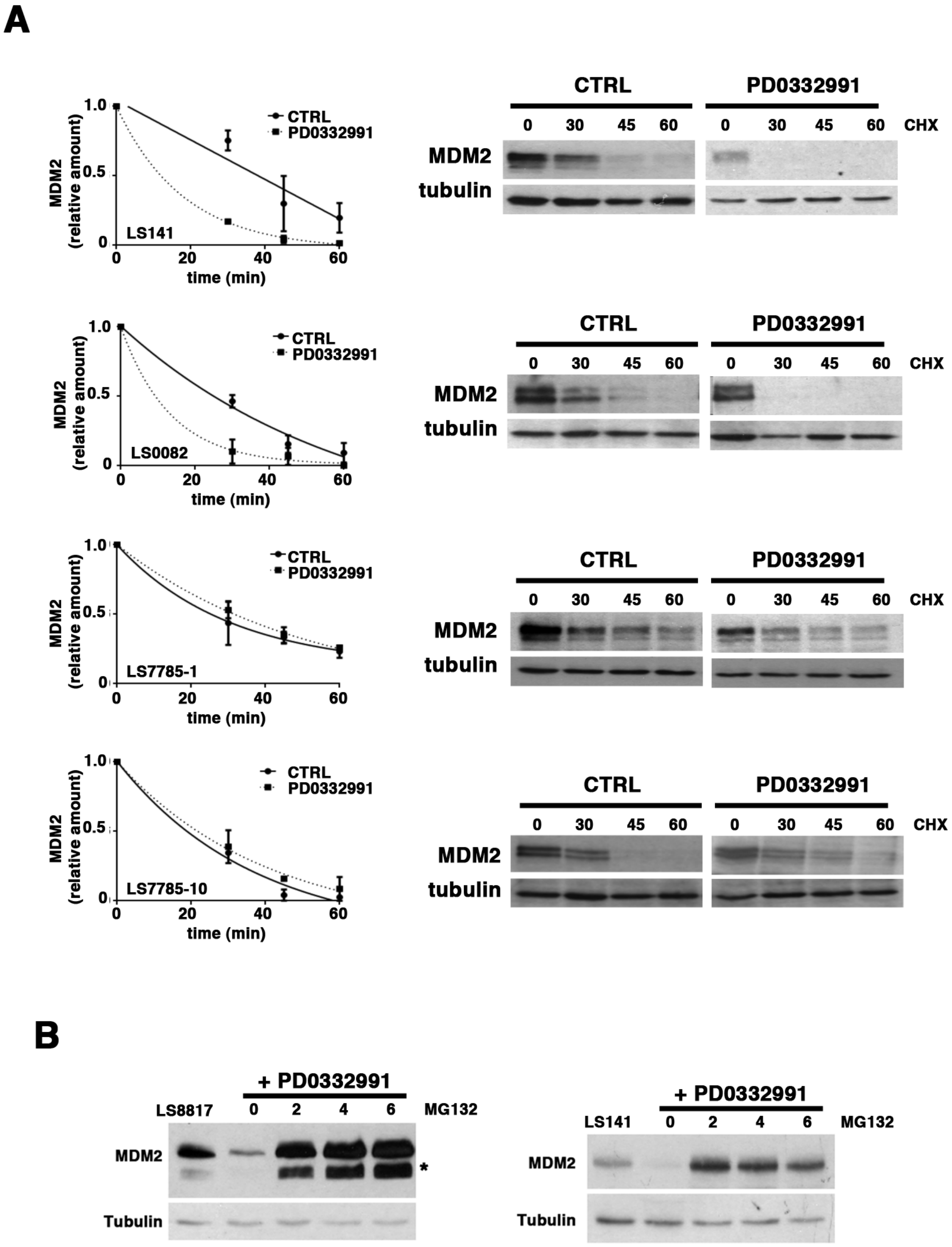

Supplementary Figure 7: MDM2 is regulated post-translationally by PD0332991 in responder cells but not non-responders. (A) The cell lines indicated were treated with 1 μM PD0332991 for two days, after which 75 μg/ml cycloheximide was added and the

amount of MDM2 measured by immunoblot at the indicated times. Tubulin is a loading control. Representative autoradiograms are shown on the right and the half-life data compiled from at least three independent experiments and plotted on the left (mean and standard deviation). (B) The cell lines indicated were treated with PD0332991 for two days after which 10μM of the proteasome inhibitor MG132 was added for the indicated times (hours). Tubulin was a loading control. This experiment was repeated twice with similar results.

A

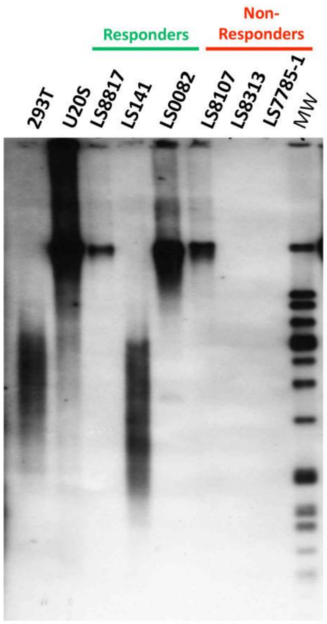

	Cell Line	TRF-predicted status
	293T	Not ALT (negative control)
	U2OS	ALT^{+} (positive control)
Responders	LS8817	ALT^{+}
Responders	LS141	Not ALT
Responders	LS0082	ALT^{+}
Non-Responders	LS8107	ALT^{+}
Non-Responders	LS8313	Not ALT
Non-Responders	LS7785-1	Not ALT

B

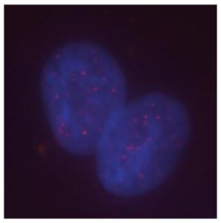

Tel-FISH ALT negative (LS8817)

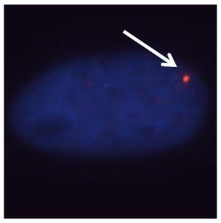

Tel-FISH ALT positive (LS8107)

	Cell Line	Cells with ALT^{+} TEL foci (%)	Tel-FISH predicted status
Responders	LS8817	0	Not ALT
Responders	LS0082	0	Not ALT
Responders	DBTRG	0	Not ALT
Responders	SNB19	0	Not ALT
Responders	MCF7	0	Not ALT
Non-Responders	LS8107	15.7	ALT^{+}
Non-Responders	LS8313	0	Not ALT
Non-Responders	LS7785-1	0	Not ALT
Non-Responders	LS7785-10	0	Not ALT

Supplementary Figure 8: Responder status does not associate with the ALT status of the cell lines. (A) The telomere restriction fragment length assay (TRF) [1] and (B) the telomere-FISH assay (tel-FISH) [2] were used to assess ALT status in the WD/DDLS cell lines. Representative examples are shown on the left and the outcome tabulated on

the right. In the tel-FISH assay the arrow points to the large “dot” indicative of ALT whereas the smaller distributed dots are indicative of a non-ALT cell.

1. Engelhardt M, Ozkaynak MF, Drullinsky P, Sandoval C, Tugal O, Jayabose S and Moore MA. Telomerase activity and telomere length in pediatric patients with malignancies undergoing chemotherapy. Leukemia. 1998; 12(1):13-24.
2. Lovejoy CA, Li W, Reisenweber S, Thongthip S, Bruno J, de Lange T, De S, Petrini JH, Sung PA, Jasin M, Rosenbluh J, Zwang Y, Weir BA, Hatton C, Ivanova E, Macconaill L, et al. Loss of ATRX, genome instability, and an altered DNA damage response are hallmarks of the alternative lengthening of telomeres pathway. PLoS Genet. 2012; 8(7):e1002772.
